# Dosimetric Comparison of Five Radiotherapy Techniques in Left‐Sided Breast Cancer Patients Following Breast‐Conserving Surgery

**DOI:** 10.1155/tbj/6666976

**Published:** 2026-10-13

**Authors:** Yanqiu Zhang, A’meng Han, Sen Yang, Jianxin Zhao, Xiumei Li

**Affiliations:** ^1^ Department of Radiotherapy First Hospital of Qinhuangdao Qinhuangdao 066000 Hebei China qhdsdyyy.com; ^2^ State Key Laboratory of Metastable Materials Science and Technology Yanshan University Qinhuangdao 066004 Hebei China ysu.edu.cn

**Keywords:** breast-conserving surgery cancer patients, CRT, CRT + IMRT, CRT + VMAT, dosimetric, IMRT, VMAT

## Abstract

**Objective:**

This study aims to compare and evaluate the dosimetric characteristics of five radiotherapy techniques in left‐sided breast cancer patients following breast‐conserving surgery. The five radiotherapy techniques include three‐dimensional conformal radiation therapy (3D‐CRT), fixed‐field intensity‐modulated radiation therapy (ff‐IMRT), volumetric modulated arc therapy (VMAT), hybrid 3D‐CRT with IMRT (CRT + IMRT), and hybrid 3D‐CRT with VMAT (CRT + VMAT).

**Patients and Methods:**

Ten patients with left‐sided breast cancer who received postlumpectomy radiotherapy at the Department of Radiation Oncology, First Hospital of Qinhuangdao, between June 1, 2020, and June 1, 2021, were randomly enrolled in this study. For each patient, five distinct treatment plans were designed using the five aforementioned techniques (3D‐CRT, ff‐IMRT, VMAT, CRT + IMRT, and CRT + VMAT) based on identical computed tomography (CT) imaging datasets. Dosimetric parameters for the tumor target volume, organs at risk (OARs), and normal tissues (defined as body minus planning target volume [PTV], termed B‐P) were analyzed and compared using dose–volume histograms (DVHs) and isodose distributions.

**Results:**

The CRT + VMAT technique demonstrated superior PTV dose coverage, conformity, and homogeneity compared to 3D‐CRT, ff‐IMRT, VMAT, and CRT + IMRT (*p* < 0.05). In contrast, the VMAT technique achieved significantly lower V_30_ (%) values for bilateral lungs and ipsilateral lung than 3D‐CRT, ff‐IMRT, CRT + IMRT, and CRT + VMAT (*p* < 0.05). The CRT + IMRT technique reduced V_5_ (%) values for bilateral lungs, ipsilateral lung, and heart compared to 3D‐CRT, ff‐IMRT, VMAT, and CRT + VMAT (*p* < 0.05). Finally, 3D‐CRT required the fewest monitor units (MUs) and shortest treatment time among all techniques (*p* < 0.05).

**Conclusion:**

Clinicians may tailor radiotherapy plans to individual patients based on the distinct dosimetric profiles of each technique. For patients requiring optimal PTV dose distribution, the CRT + VMAT technique is suggest. For enhanced cardiopulmonary protection (e.g., reduced lung/heart doses), ff‐IMRT or CRT + IMRT may be prioritized. Additionally, 3D‐CRT should be the first choice for patients needing rapid fractionated treatment due to its efficiency in minimizing MUs and treatment time.

**Statement of Significance**
 The International Agency for Cancer Research of the World Health Organization released the latest global cancer burden data in 2020, which indicated that breast cancer had replaced lung cancer as the number one cancer in the world. Routine radiotherapy after breast‐conserving surgery could reduce the local recurrence rate and distant metastasis rate and improve the overall survival rate. Therefore, in‐depth study of radiotherapy technology for breast cancer was of great significance. The postoperative radiotherapy technologies for breast cancer mainly included 3D‐CRT, ff‐IMRT, VMAT, and combined radiotherapy technologies: CRT + IMRT and CRT + VMAT. A comprehensive study and analysis of the dosimetric characteristics of these five radiotherapy techniques had important clinical significance for guiding how to choose radiotherapy techniques after breast cancer surgery.

## 1. Introduction

Postoperative radiotherapy is a standard component of breast‐conserving therapy for early‐stage breast cancer, significantly improving both local control and overall survival [1]. For patients with left‐sided breast cancer, various advanced radiotherapy techniques are available, including conformal radiation therapy (CRT), intensity‐modulated radiation therapy (IMRT), and volumetric modulated arc therapy (VMAT), as well as hybrid approaches such as CRT + IMRT and CRT + VMAT [2–9].

While these techniques aim to enhance target coverage and spare organs at risk (OARs), there is a lack of comprehensive dosimetric comparisons among all five modalities. Most previous studies have only evaluated two or three techniques [10–12], leaving a gap in the evidence needed to guide clinical decision‐making. This gap is particularly relevant, given the long survival expectancy of these patients, where reducing radiation dose to OARs, particularly for the heart and ipsilateral lung, is critical for minimizing late toxicities such as cardiac events and radiation pneumonitis, thereby preserving long‐term quality of life.

Although CRT using tangential fields has been widely used, it often results in suboptimal dose homogeneity and conformity. More modern techniques such as IMRT and VMAT offer improved dosimetric quality, yet their relative merits in a unified comparison remain unclear.

Therefore, this study aims to perform a systematic dosimetric comparison of CRT, IMRT, VMAT, CRT + IMRT, and CRT + VMAT in left‐sided breast cancer. We seek to characterize the advantages and limitations of each technique, with a focus on OAR sparing and target quality, in order to provide evidence‐based guidance for their clinical application.

## 2. Materials and Methods

### 2.1. Patient Selection

This study retrospectively enrolled 10 female patients with left‐sided breast cancer who underwent breast‐conserving surgery followed by radiotherapy at our institution’s Department of Radiation Oncology between June 1, 2020, and June 1, 2021. All patients were female, aged 26–65 years (median: 46 years), with Karnofsky Performance Status (KPS) scores ranging from 75 to 92. The planning target volume (PTV) for each patient measured 250.9–943.7 cm^3^. One patient (Case No. 02) had Stage 0 ductal carcinoma in situ with a PTV volume of only 250.9 cc, which was substantially smaller than the second smallest volume (409.7 cc). Detailed baseline clinical characteristics are summarized in Table 1.

**TABLE 1:** General clinical data of patients.

Case no.	Sex	Age (yeas)	T stage	N stage	M stage	Clinical stage	Pathological pattern	PTV volume (cc)
01	Female	65	1	0	0	I	Invasive ductal carcinoma	767.5
02	Female	36	is	0	0	0	Ductal carcinoma in situ	250.9
03	Female	41	1	0	0	I	Invasive adenocarcinoma	943.7
04	Female	41	1	0	0	I	Invasive lobular carcinoma	460.3
05	Female	64	1	0	0	I	Invasive ductal carcinoma	868.5
06	Female	64	1	1	0	IIA	Invasive ductal carcinoma	654.5
07	Female	39	is	0	0	0	Ductal carcinoma in situ	409.7
08	Female	50	1	0	0	I	Invasive ductal carcinoma	621.2
09	Female	44	2	1	0	IIB	Invasive ductal carcinoma	656.4
10	Female	62	1	0	0	I	Invasive ductal carcinoma	692.3

### 2.2. Position Simulation

Patients were positioned supine on a breast board mounted on the computed tomography (CT) couch, with arms raised overhead and the sternum aligned parallel to the couch. The supervising radiation oncologist placed surface markers to delineate the breast, surgical scar, and skin fiducials. Simulation CT scans were acquired using a GE Healthcare CT scanner, covering from the hyoid bone level to the upper abdomen, with a slice thickness and spacing of 2.5 mm. The CT images were transferred to the Varian ARIA Oncology Information System via the ARIA server and subsequently accessed within the treatment planning system (TPS) for contouring and plan design.

### 2.3. Targets and OARs

Target volumes and OARs were contoured by two senior radiation oncologists. The clinical target volume (CTV) was defined to encompass the entire left breast and pectoralis major fascia, while excluding the skin, pectoralis minor muscle, ribs, and intercostal spaces unless directly involved by disease. The PTV was generated by expanding the CTV with anisotropic margins: superior–inferior direction: 1.0–1.5 cm, medial–lateral (sternal/axillary) and anterior–posterior (lung interface) directions: 0.5–1.0 cm, skin surface: no margin was added (limited to 0.5 cm beneath the skin). OARs included the heart, ipsilateral lung, bilateral lungs, esophagus, contralateral breast, spinal cord, and skin.

### 2.4. Treatment Plan Design

All treatment plans were designed by senior medical physicists using the Varian Eclipse TPS (Version 15.6). For each patient, five distinct plans were developed on the same CT dataset. Common parameters across all plans included 6 MV x‐ray beams with identical isocenter positioning, anisotropic analytical algorithm (AAA) for dose calculation, and optimization and calculation grid sizes of 0.25 cm. All plans were optimized for delivery on a Varian Trilogy linear accelerator. Jaw tracking was not enabled; the standard dynamic MLC mode without jaw tracking was used for all techniques. The MLC was the Millennium 120‐leaf MLC with a leaf width of 5 mm (central 20 cm) and 10 mm (outer leaves). Schematic diagrams of the five techniques are illustrated in Figures 1, 2, 3, 4, and 5, with their key characteristics summarized in Table 2. Prescription and dose constraints: A total dose of 50 Gy in 25 fractions (2 Gy per fraction) was prescribed for all plans. A 4:1 dose contribution ratio was maintained between 3D‐CRT and ff‐IMRT/VMAT. The 4:1 dose contribution ratio (80% from 3D‐CRT and 20% from IMRT or VMAT) was adopted from a previously published hybrid planning study for left‐sided breast cancer [13]. Dose constraints, per NCCN guidelines and prior studies, were defined as follows: for all techniques, optimization objectives included target coverage (PTV D_95%_ ≥ 95%, D_max_ ≤ 110% of prescription, D_min_ ≥ 90%) and high‐dose OAR constraints (spinal cord D_max_ < 40 Gy, esophagus D_mean_ < 20 Gy, heart D_mean_ < 10 Gy, ipsilateral lung V_20_ < 30%). No explicit constraints or penalties were applied to low‐dose volumes (e.g., V_5_, V_10_) for any technique. The number of segments/control points for IMRT and VMAT was as follows: for ff‐IMRT: 4 fields (two fields at angles identical to 3D‐CRT and two additional fields offset by 20°), with a maximum of 30 segments per field (average 25 segments per field); for VMAT: two partial arcs, with a maximum of 178 control points per arc (total 356 control points); and for CRT + IMRT and CRT + VMAT hybrid plans, the same segmentation/control point settings were used for the IMRT/VMAT components.

**FIGURE 1:**
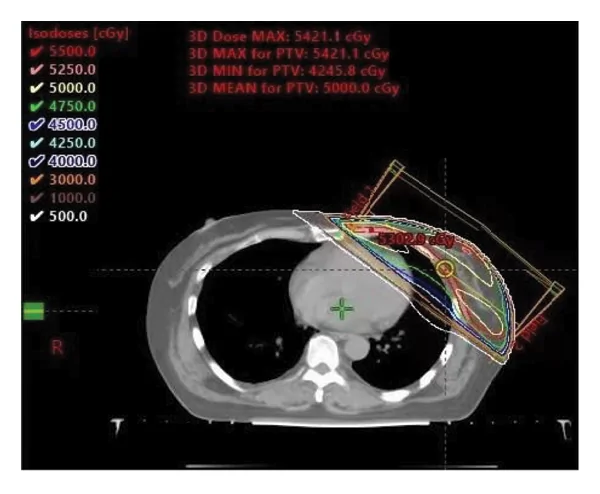
Schematic diagrams of 3D‐CRT.

**FIGURE 2:**
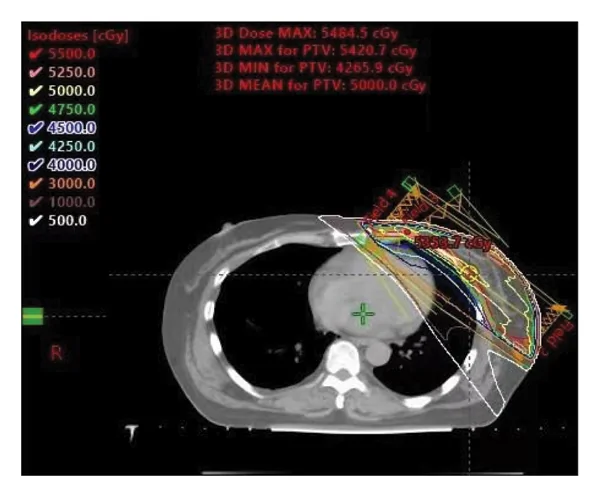
Schematic diagrams of ff‐IMRT.

**FIGURE 3:**
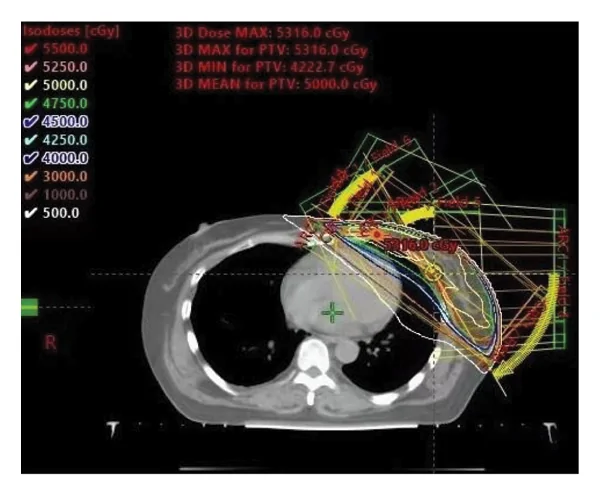
Schematic diagrams of VMAT.

**FIGURE 4:**
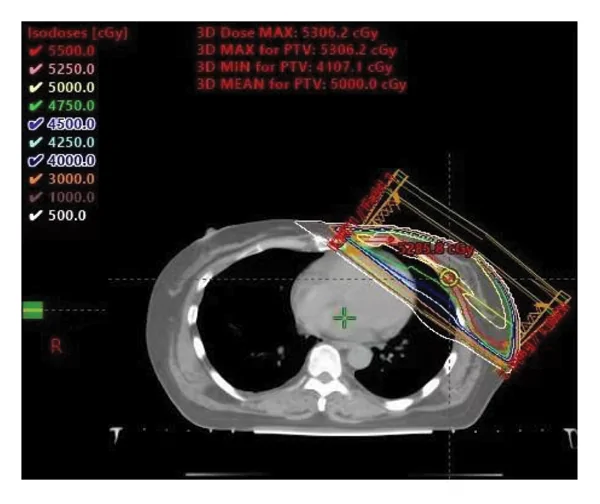
Schematic diagrams of CRT + IMRT.

**FIGURE 5:**
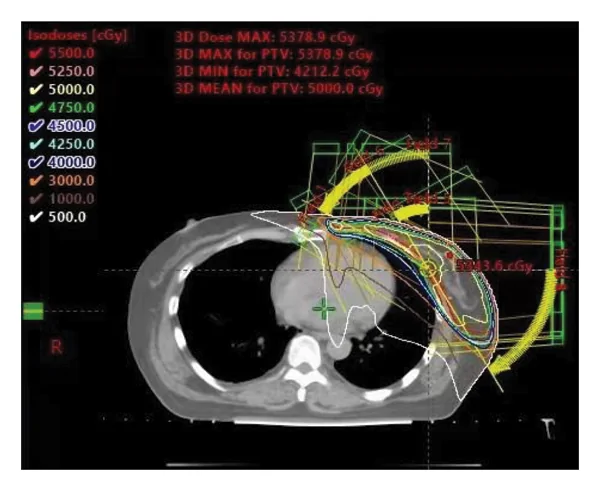
Schematic diagrams of CRT + VMAT.

**TABLE 2:** Technical characteristics of the five radiotherapy techniques.

	3D‐CRT	Ff‐IMRT	VMAT	CRT + IMRT	CRT + VMAT
Number of fields/arcs	2 (with wedges)	4 (fixed fields)	4 (partial arcs)	2 (conformal fields) + 2 (fixed fields))	2 (conformal fields) + 2 (partial arcs)

Field/arc angles	Two opposing tangential fields were used. Gantry angles were individually optimized for each patient based on the breast shape and chest wall curvature, typically ranging from 300° to 320° for the medial field and 140° to 160° for the lateral field	Two fields at angles identical to 3D‐CRT; two additional fields offset by 20°	Arc 1: Start angle matches 3D‐CRT first field; stop angle matches 3D‐CRT second field. Arc 2: Opposite direction with the same angular range. Both arcs avoid 120° midarc segments.	CRT/IMRT fields aligned with 3D‐CRT angles	CRT fields aligned with 3D‐CRT angles; VMAT arc matches angular range of Technique 3 (VMAT)

Collimator angles	Parallel to target, minimizing lung intersection	Parallel to target, minimizing lung intersection	10°or 350°	Parallel to target, minimizing lung intersection	CRT: parallel to target, minimizing lung intersection. VMAT: 10° or 350°

### 2.5. Evaluation Methods

Isodose distributions and dose–volume histograms (DVHs) (as shown in Figure 6) of the five techniques were compared to determine dosimetric parameters for the target volume, OARs, and normal tissues (defined as body minus PTV, termed B‐P). The target volume was evaluated using the following metrics: minimum dose, maximum dose, median dose, homogeneity index (HI) [14], conformity index (CI) [15], and volume receiving ≥ 105% of the prescription dose.

**FIGURE 6:**
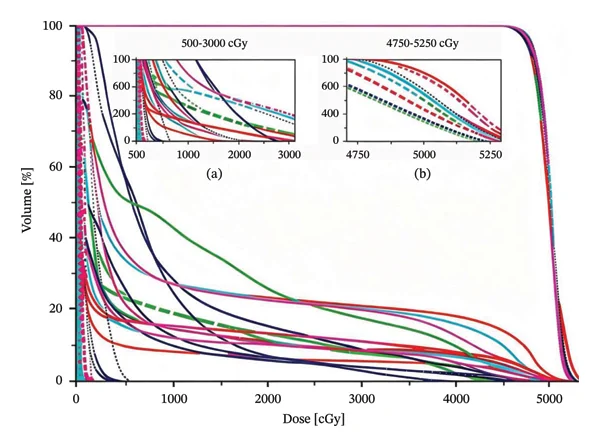
Dose–volume histograms (DVHs) of the five techniques. DVH comparison of five techniques using color and line styles: 3D‐CRT (red, solid), ff‐IMRT (green, dashed), VMAT (blue, dotted), CRT + IMRT (cyan, dashed‐dotted), CRT + VMAT (magenta, dashed‐dotted‐dotted). Insets zoom into 5–30 Gy (left) and 4750–5250 Gy (right). Dual coding enhances readability in color and grayscale.

The HI and CI were calculated using the following equations:

• Homogeneity Index (HI)
(1) HI=D2%−D98%Dprescription×100%,
 where D_2%_ is the maximum dose received by 2% of the target volume, D_98%_ is the minimum dose received by 98% of the target volume, and D_prescription_ is the prescription dose.
• Conformity Index (CI):
(2) CI=Vt,refVt×Vt,refVref,
 where V_t_ is the volume of the PTV, V_t,ref_ is the volume of the PTV covered by the reference isodose line, and V_ref_ is the total volume covered by the reference isodose line.

The following parameters were used to evaluate OARs: the maximum dose of spinal cord (D_max_); the mean dose of esophagus (D_mean_); V_10_, V_20_, and V_30_ of heart; V_5_, V_10_, V_20_, and V_30_ of lungs and ipsilateral lung; the maximum dose and D_5_ of contralateral lung; the mean dose and D_5_ of contralateral breast; and the mean dose and D_5_ of skin. V_x_: percentage of the organ volume receiving at least x Gy; the Dx here refers to the dose delivered to x% of the organ volume. For normal tissues (B‐P), the percentage of the volume receiving 5–50 Gy was calculated. Additionally, the monitor units (MUs) required for each plan were compared to assess treatment efficiency.

A preplanned subgroup analysis was performed to investigate the potential effect of PTV volume on the comparative performance of the five techniques. The cohort was stratified into two subgroups based on the median PTV volume (655.45 cc): a “small PTV” group (< 655.45 cc, *n* = 5) and a “large PTV” group (≥ 655.45 cc, *n* = 5). All primary dosimetric endpoints were reanalyzed independently within each subgroup using the same statistical procedures as for the entire cohort.

### 2.6. Statistical Analysis

Statistical analyses were performed using SPSS 22.0 software (IBM Corp., Armonk, NY, USA). The normality of the five datasets was confirmed via the Kolmogorov–Smirnov test, followed by paired *t* tests to compare dosimetric differences across the five plans. A two‐tailed *p* value < 0.05 was considered statistically significant. Pairwise comparisons between the five techniques were performed using paired *t* tests. No adjustment for multiple comparisons (e.g., Bonferroni correction) was applied; therefore, the results should be interpreted as exploratory, and *p* values between 0.01 and 0.05 are described as indicating a trend “toward” rather than definitive significance.

## 3. Results

The dosimetric comparison revealed distinct patterns of dose distribution among the five techniques, with significant implications for clinical decision‐making. The results are synthesized below, prioritizing OAR sparing and its link to clinical outcomes. The detailed results are presented in Tables 2 and 3. *p* values obtained from paired‐samples *t* test for multiple comparisons are summarized in Table 4.

**TABLE 3:** Summary of the dosimetric parameters of PTV, OAR and B‐P.

Parameter	CRT	IMRT	VMAT	CRT + IMRT	CRT + VMAT
*PTV*					
D_min_ (cGy)	4067.01 ± 198.45	4155.27 ± 196.09	4104.62 ± 132.16	4056.87 ± 178.57	4158.33 ± 131.03
D_max_ (cGy)	5389.66 ± 47.54	5422.87 ± 67.84	5390.07 ± 48.42	5311.84 ± 121.67	5307.86 ± 54.48
D_median_ (cGy)	5012.98 ± 12.62	4983.15 ± 66.13	5032.22 ± 47.55	5028.43 ± 8.20	5045.65 ± 60.32
CI	0.57 ± 0.05	0.77 ± 0.06	0.80 ± 0.04	0.70 ± 0.08	0.79 ± 0.05
HI	0.26 ± 0.04	0.24 ± 0.04	0.24 ± 0.03	0.24 ± 0.04	0.21 ± 0.02
V_105%_ (cc)	46.42 ± 40.88	15.81 ± 26.64	17.90 ± 41.83	7.44 ± 16.34	17.21 ± 40.54

*CL lung*					
D_max_ (cGy)	2071.53 ± 2126.95	1668.11 ± 1810.63	1072.93 ± 478.79	1887.48 ± 2193.64	1109.55 ± 1395.25
clungD_5%_ (Gy)	1453.95 ± 2254.93	1177.41 ± 1853.80	466.32 ± 286.68	1832.67 ± 2309.92	1055.56 ± 1793.85

*Lungs*					
V_30Gy_ (%)	8.93 ± 2.53	7.43 ± 1.63	6.99 ± 1.67	8.47 ± 2.34	8.63 ± 2.29
V_20Gy_ (%)	9.89 ± 2.61	10.43 ± 1.78	10.78 ± 2.30	9.60 ± 2.47	9.80 ± 2.42
V_10Gy_ (%)	11.55 ± 2.77	16.90 ± 2.44	18.12 ± 3.83	11.27 ± 2.60	12.18 ± 2.49
V_5Gy_ (%)	14.49 ± 3.20	24.71 ± 4.10	30.62 ± 7.35	14.07 ± 2.98	17.51 ± 3.02

*IL lung*					
V_30Gy_ (%)	20.23 ± 5.40	16.85 ± 3.50	15.87 ± 3.64	19.19 ± 5.01	19.55 ± 4.86
V_20Gy_ (%)	22.41 ± 5.55	23.69 ± 3.96	24.47 ± 4.99	21.77 ± 5.26	22.20 ± 5.12
V_10Gy_ (%)	26.16 ± 5.87	38.34 ± 5.17	40.06 ± 7.00	25.55 ± 5.52	27.60 ± 5.12
V_5Gy_ (%)	32.82 ± 6.76	53.12 ± 7.70	60.95 ± 9.16	31.87 ± 6.33	39.47 ± 5.83

*Heart*					
V_30Gy_ (%)	9.63 ± 2.54	5.57 ± 2.08	5.05 ± 3.42	8.60 ± 2.47	8.82 ± 2.54
V_20Gy_ (%)	11.36 ± 2.65	9.22 ± 2.80	10.66 ± 6.20	10.61 ± 2.58	10.81 ± 2.75
V_10Gy_ (%)	13.89 ± 2.74	18.41 ± 3.42	29.04 ± 15.57	13.35 ± 2.69	14.05 ± 3.05

*Esophagus*					
D_mean_ (cGy)	45.48 ± 8.16	78.21 ± 46.73	224.79 ± 50.63	42.92 ± 7.39	110.71 ± 41.71

*Spinal cord*					
D_max_ (cGy)	36.40 ± 8.66	49.10 ± 15.94	305.88 ± 138.23	33.06 ± 10.58	139.10 ± 104.93

*CL breast*					
D_mean_ (cGy)	40.88 ± 60.38	130.13 ± 81.16	188.90 ± 109.74	35.97 ± 52.22	122.28 ± 48.20
D_5_ (cGy)	78.90 ± 88.46	337.46 ± 166.26	415.03 ± 150.21	155.43 ± 269.55	242.37 ± 238.29

*Skin*					
D_mean_ (cGy)	520.63 ± 192.39	566.47 ± 55.81	554.40 ± 107.25	550.74 ± 67.83	563.50 ± 71.69
D_5_ (cGy)	3978.78 ± 1394.78	3958.59 ± 1389.51	4056.52 ± 289.27	4342.21 ± 118.55	4282.94 ± 186.17

*B-P*					
V_5Gy_ (%)	8.74 ± 1.16	15.33 ± 2.42	18.70 ± 2.96	9.18 ± 2.65	11.12 ± 2.71
V_10Gy_ (%)	7.46 ± 1.00	9.21 ± 1.42	11.20 ± 1.55	7.90 ± 2.62	8.80 ± 3.09
V_15Gy_ (%)	6.69 ± 0.94	6.72 ± 0.94	8.09 ± 1.13	7.11 ± 2.47	7.78 ± 3.02
V_20Gy_ (%)	6.04 ± 0.90	5.46 ± 0.78	6.25 ± 0.85	6.45 ± 2.39	7.08 ± 2.89
V_25Gy_ (%)	5.58 ± 0.86	4.88 ± 1.15	4.94 ± 0.64	5.93 ± 2.18	6.51 ± 2.60
V_30Gy_ (%)	5.24 ± 0.83	3.88 ± 0.53	3.89 ± 0.48	5.45 ± 1.92	5.96 ± 2.25
V_35Gy_ (%)	4.88 ± 0.79	3.15 ± 0.44	2.96 ± 0.40	4.87 ± 1.64	5.28 ± 1.87
V_40Gy_ (%)	4.35 ± 0.73	2.27 ± 0.47	1.98 ± 0.42	3.97 ± 1.30	4.12 ± 1.49
V_45Gy_ (%)	3.20 ± 0.59	1.20 ± 0.38	0.89 ± 0.39	2.36 ± 0.90	2.16 ± 1.27
V_50Gy_ (%)	0.54 ± 0.29	0.15 ± 0.07	0.04 ± 0.09	0.16 ± 0.12	0.19 ± 0.27

*Delivery efficiency*					
MU	242 ± 10	1365 ± 190	451 ± 88	566 ± 35	343 ± 145
Time (s)	24 ± 1	137 ± 19	45 ± 9	57 ± 36	34 ± 15

**TABLE 4:** *p* values for multiple comparisons using paired‐samples *t* test.

Parameter	CRT vs. IMRT	CRT vs. VMAT	CRT vs. CRT + IMRT	CRT vs. CRT + VMAT	IMRT vs. VMAT	IMRT vs. CRT + IMRT	IMRT vs. CRT + VMAT	VMAT vs. CRT + IMRT	VMAT vs. CRT + VMAT	CRT + IMRT vs. CRT + VMAT
*PTV*										
D_min_	0.103	0.613	0.678	0.063	0.502	0.028^∗^	0.933	0.461	0.266	0.007^∗^
D_max_	0.224	0.988	0.092	0.006^∗^	0.294	0.014^∗^	0.010^∗^	0.097	0.003^∗^	0.936
D_median_	0.226	0.236	0.008^∗^	0.124	0.084	0.075	0.046^∗^	0.811	0.078	0.412
CI	< 0.001^∗^	< 0.001^∗^	0.001^∗^	< 0.001^∗^	0.092	0.004^∗^	0.334	< 0.001^∗^	0.301	0.001^∗^
HI	0.203	0.431	0.147	0.008^∗^	0.757	0.791	0.115	0.958	0.028^∗^	0.023^∗^
V_105%_	0.109	0.167	0.018^∗^	0.161	0.901	0.401	0.936	0.488	0.899	0.525

*CL lung*										
D_max_	0.582	0.177	0.860	0.155	0.349	0.841	0.484	0.270	0.936	0.245
D5% (Gy)	0.732	0.214	0.731	0.639	0.268	0.569	0.895	0.105	0.334	0.215

*Lungs*										
V_30Gy_	0.005^∗^	0.026^∗^	0.001^∗^	0.008^∗^	0.420	0.014^∗^	0.007^∗^	0.061	0.032^∗^	0.051
V_20Gy_	0.462	0.314	0.002^∗^	0.265	0.621	0.237	0.355	0.184	0.238	0.010^∗^
V_10Gy_	< 0.001^∗^	< 0.001^∗^	0.011^∗^	0.029^∗^	0.298	< 0.001^∗^	< 0.001^∗^	< 0.001^∗^	< 0.001^∗^	0.004^∗^
V_5Gy_	< 0.001^∗^	< 0.001^∗^	0.003^∗^	0.002^∗^	< 0.001^∗^	< 0.001^∗^	< 0.001^∗^	< 0.001^∗^	< 0.001^∗^	0.001^∗^

*IL lung*										
V_30Gy_	0.005^∗^	0.025^∗^	< 0.001^∗^	0.007^∗^	0.419	0.013^∗^	0.006^∗^	0.061	0.032^∗^	0.049^∗^
V_20Gy_	0.445	0.308	0.002^∗^	0.249	0.629	0.231	0.339	0.181	0.232	0.010^∗^
V_10Gy_	< 0.001^∗^	0.001^∗^	0.010^∗^	0.026^∗^	0.490	< 0.001^∗^	< 0.001^∗^	< 0.001^∗^	< 0.001^∗^	0.004^∗^
V_5Gy_	< 0.001^∗^	< 0.001^∗^	0.002^∗^	0.002^∗^	0.006^∗^	< 0.001^∗^	< 0.001^∗^	< 0.001^∗^	< 0.001^∗^	0.001^∗^

*Heart*										
V_30Gy_	< 0.001^∗^	< 0.001^∗^	0.012^∗^	0.011^∗^	0.584	< 0.001^∗^	< 0.001^∗^	0.001^∗^	< 0.001^∗^	0.119
V_20Gy_	0.035^∗^	0.645	0.028^∗^	0.046^∗^	0.458	0.098	0.089	0.972	0.912	0.195
V_10Gy_	0.006^∗^	0.009^∗^	0.085	0.596	0.056	0.004^∗^	0.012^∗^	0.007^∗^	0.009^∗^	0.046^∗^

*Esophagus*										
D_mean_	0.040^∗^	< 0.001^∗^	0.003	0.001^∗^	< 0.001^∗^	0.030^∗^	0.182	< 0.001^∗^	< 0.001^∗^	0.001^∗^

*Spinal cord*										
D_max_	0.004^∗^	< 0.001^∗^	0.059	0.011^∗^	< 0.001^∗^	< 0.001^∗^	0.019^∗^	< 0.001^∗^	0.021^∗^	0.008^∗^

*CL breast*										
D_mean_	0.001^∗^	0.009^∗^	0.124	0.002^∗^	0.168	0.001^∗^	0.795	0.006^∗^	0.145	0.001^∗^
D5% (Gy)	< 0.001^∗^	< 0.00^∗^	0.423	0.076	0.155	0.032^∗^	0.183	0.018^∗^	0.049^∗^	0.001^∗^

*Skin*										
D_mean_	0.454	0.367	0.588	0.444	0.645	0.104	0.725	0.864	0.658	0.014^∗^
D5% (Gy)	0.619	0.879	0.448	0.537	0.847	0.423	0.509	0.001^∗^	0.001^∗^	0.110

*B-P*										
V_5Gy_	< 0.001^∗^	< 0.001^∗^	0.549	0.019^∗^	0.001	0.001^∗^	0.007^∗^	< 0.001^∗^	< 0.001^∗^	0.014^∗^
V_10Gy_	0.001^∗^	< 0.001^∗^	0.552	0.194	< 0.001^∗^	0.129	0.682	0.011^∗^	0.064	0.231
V_15Gy_	0.900	0.003^∗^	0.554	0.269	< 0.001^∗^	0.570	0.256	0.299	0.779	0.343
V_20Gy_	0.052	0.446	0.558	0.277	0.001	0.173	0.104	0.806	0.426	0.357
V_25Gy_	0.057	0.014^∗^	0.590	0.288	0.860	0.023^∗^	0.042^∗^	0.186	0.107	0.352
V_30Gy_	< 0.001^∗^	< 0.001^∗^	0.707	0.341	0.893	0.018^∗^	0.016^∗^	0.026^∗^	0.020^∗^	0.339
V_35Gy_	< 0.001^∗^	< 0.001^∗^	0.975	0.524	0.107	0.005^∗^	0.006^∗^	0.004^∗^	0.004^∗^	0.336
V_40Gy_	< 0.001^∗^	< 0.001^∗^	0.334	0.655	0.059	0.002^∗^	0.005^∗^	0.001^∗^	0.001^∗^	0.657
V_45Gy_	< 0.001^∗^	< 0.001^∗^	0.010^∗^	0.034^∗^	0.036^∗^	0.002^∗^	0.043^∗^	< 0.001^∗^	0.007^∗^	0.524
V_50Gy_	0.004^∗^	0.001^∗^	0.002^∗^	0.019^∗^	0.006^∗^	0.813	0.611	0.025^∗^	0.060	0.630

*Delivery efficiency*										
Mu	< 0.001^∗^	< 0.001^∗^	< 0.001^∗^	0.056	< 0.001^∗^	< 0.001^∗^	< 0.001^∗^	0.005^∗^	0.015^∗^	0.001^∗^
Time (s)	< 0.001^∗^	< 0.001^∗^	< 0.001^∗^	0.061	< 0.001^∗^	< 0.001^∗^	< 0.001^∗^	0.004^∗^	0.014^∗^	0.001^∗^

^∗^Indicates statistical significance.

### 3.1. Cardiac Sparing: A Critical Trade‐Off

Given the paramount importance of minimizing late cardiac toxicity in left‐sided breast cancer patients, heart dose was a primary endpoint. Our analysis revealed a clear trade‐off between high‐ and low‐dose exposure.

• High‐Dose Reduction (V_30Gy_/V_20Gy_): The modulated techniques (IMRT, VMAT, CRT + IMRT, CRT + VMAT) consistently and significantly reduced volumes of the heart receiving high doses (V_30Gy_, V_20Gy_) compared to CRT (all *p* < 0.05). Among them, VMAT provided the most robust reduction in V_30Gy_, outperforming even the other advanced modalities.
• Low‐Dose Bath (V_10Gy_): Conversely, CRT demonstrated a significant advantage in the low‐dose region, with its V_10Gy_ being lower than all other techniques (all *p* < 0.01). This creates a critical clinical choice: prioritizing the reduction of high‐dose volumes (linked to perfusion defects and major cardiac events) with advanced techniques, versus accepting a higher low‐dose bath.

### 3.2. Pulmonary Sparing: Balancing Toxicity Risks

Sparing the lungs is crucial to mitigate the risk of radiation pneumonitis and preserve pulmonary function.

• Intermediate‐/High‐Dose Volumes (V_30Gy_): The hybrid technique CRT + IMRT was most effective at minimizing the V_30Gy_ of the ipsilateral lung, a key predictor for symptomatic pneumonitis, showing significant improvement over CRT, IMRT, and VMAT.
• Low‐Dose Exposure (V_5Gy_/V_10Gy_): As seen with the heart, CRT plans resulted in the lowest low‐dose volumes (V_5Gy_/V_10Gy_) to the lungs. In contrast, IMRT and VMAT plans generated the most extensive low‐dose bath, a factor potentially relevant for long‐term lung function and secondary malignancy risk.

### 3.3. Target Dosimetry and Coverage

• All advanced techniques provided significantly superior target conformity (CI) compared to CRT (*p* < 0.001). CRT + VMAT yielded the most homogeneous dose distribution (best HI), significantly better than CRT, IMRT, and CRT + IMRT. This suggests that for targets where precise conformation and dose uniformity are the highest priority, CRT + VMAT may be a favorable option.

### 3.4. Dose to Other OARs and Body‐Plexus

• Esophagus, Spinal Cord, and Contralateral Breast: CRT plans delivered the lowest doses to these OARs. The higher doses associated with IMRT/VMAT, while still within constraints, suggest that CRT may be preferable when these structures are of particular concern.
• Body‐Plexus (B‐P): A consistent pattern emerged: CRT minimized low‐dose volumes (V_5–15Gy_) but resulted in larger high‐dose volumes (V_40–50Gy_). IMRT and VMAT exhibited the inverse. This underscores the fundamental dosimetric trade‐off between modern and conventional techniques.

### 3.5. Treatment Delivery Efficiency

In addition to dosimetric quality, the efficiency of treatment delivery was evaluated, as it directly impacts clinical workflow, patient comfort, and resource utilization. The analysis of MUs and estimated beam‐on time revealed distinct differences among the techniques. Key findings are presented in Tables 2 and 3.

CRT was the most efficient technique, requiring the lowest number of MUs. Among the advanced modalities, VMAT was the most efficient, demonstrating a significant reduction in both MUs and estimated treatment time compared to IMRT and the hybrid techniques (CRT + IMRT and CRT + VMAT). IMRT required the highest number of MUs, correlating with longer delivery times. The hybrid techniques showed intermediate efficiency, with their delivery times and MUs falling between those of CRT and the full modulation techniques.

These findings highlight a key clinical trade‐off: While IMRT and hybrid techniques can offer superior dose conformity and OAR sparing in specific scenarios, VMAT provides a favorable balance of delivery efficiency and dosimetric quality, which is a crucial consideration in high‐throughput clinical environments.

### 3.6. Subgroup Analysis Based on PTV Volume

The analysis revealed that the overall dosimetric superiority of the advanced techniques over conventional CRT for target conformity was maintained regardless of PTV size. For instance, the CI for VMAT remained significantly better than that for CRT in both the small and large PTV subgroups (*p* < 0.01 for both).

A significant volume‐dependent interaction was observed for several critical OAR parameters. For the CRT vs. CRT + IMRT comparison in the PTV volume‐based subgroup analysis, no statistically significant differences in heart and lung dose–volume parameters were detected in the small PTV group. However, these parameters reached statistical significance in the large PTV group. Detailed results for the above‐mentioned findings are shown in Tables 5 and 6.

**TABLE 5:** *p* values for multiple comparisons using paired‐samples *t* test (small‐volume subgroups).

Parameter	CRT vs. IMRT	CRT vs. VMAT	CRT vs. CRT + IMRT	CRT vs. CRT + VMAT	IMRT vs. VMAT	IMRT vs. CRT + IMRT	IMRT vs. CRT + VMAT	VMAT vs. CRT + IMRT	VMAT vs. CRT + VMAT	CRT + IMRT vs. CRT + VMAT
*PTV*										
D_min_	0.061	0.302	0.772	0.120	0.321	0.003^∗^	0.231	0.175	0.471	0.015^∗^
D_max_	0.642	0.384	0.190	0.229	0.736	0.086	0.303	0.034^∗^	0.070	0.363
D_median_	0.230	0.365	0.366	0.240	0.111	0.183	0.076	0.474	0.145	0.303
CI	< 0.001^∗^	0.004^∗^	0.049^∗^	0.003^∗^	0.193	0.075	0.145	0.015^∗^	0.717	0.003^∗^
HI	0.132	0.131	0.458	0.062	0.573	0.129	0.927	0.557	0.374	0.111
V_105%_	0.068	0.831	0.037^∗^	0.626	0.459	0.153	0.329	0.363	0.570	0.247

*CL lung*										
D_max_	0.936	0.259	0.550	0.153	0.302	0.574	0.169	0.809	0.002^∗^	0.489
D5% (Gy)	0.951	0.270	0.614	0.640	0.229	0.573	0.598	0.591	0.507	0.233

*Lungs*										
V_30Gy_	0.040^∗^	0.061	0.060	0.094	0.408	0.040^∗^	0.036^∗^	0.089	0.060	0.270
V_20Gy_	0.807	0.874	0.108	0.535	0.973	0.568	0.709	0.706	0.804	0.157
V_10Gy_	0.021^∗^	0.034^∗^	0.278	0.153	0.995	0.016^∗^	0.019^∗^	0.032^∗^	0.024^∗^	0.099
V_5Gy_	0.005^∗^	0.009^∗^	0.157	0.050	0.027^∗^	0.005^∗^	0.001^∗^	0.009^∗^	0.006^∗^	0.042^∗^

*IL lung*										
V_30Gy_	0.035^∗^	0.060	0.054	0.085	0.409	0.036^∗^	0.032^∗^	0.089	0.060	0.271
V_20Gy_	0.804	0.880	0.100	0.523	0.967	0.565	0.339	0.714	0.808	0.161
V_10Gy_	0.021^∗^	0.032^∗^	0.268	0.152	0.988	0.015^∗^	0.703	0.030^∗^	0.023^∗^	0.096
V_5Gy_	0.003^∗^	0.002^∗^	0.124	0.047^∗^	0.006^∗^	0.025^∗^	0.001^∗^	0.002^∗^	0.001^∗^	0.039^∗^

*Heart*										
V_30Gy_	0.027^∗^	0.003^∗^	0.143	0.133	0.189	0.025^∗^	0.030^∗^	0.002^∗^	0.002^∗^	0.234
V_20Gy_	0.145	0.013^∗^	0198	0.208	0.717	0.236	0.233	0.030^∗^	0.009	0.378
V_10Gy_	0.076	0.106	0.321	0.532	0.160	0.082	0.113	0.095	0.115	0.066

*Esophagus*										
D_mean_	0.149	< 0.001^∗^	0.124	0.015^∗^	< 0.001^∗^	0.129	0.050	< 0.001^∗^	0.000^∗^	0.015^∗^

*Spinal cord*										
D_max_	0.098	0.098	0.222	0.044^∗^	0.022^∗^	0.018	0.043^∗^	0.017^∗^	0.032^∗^	0.030^∗^

*CL breast*										
D_mean_	0.024	0.007^∗^	0.350	0.015^∗^	0.047^∗^	0.029^∗^	0.858	0.008^∗^	0.363	0.008^∗^
D5% (Gy)	0.021	0.002	0.348	0.088	0.002^∗^	0.029^∗^	0.142	0.029^∗^	0.006^∗^	0.014^∗^

*Skin*										
D_mean_	0.402	0.384	0.462	0.392	0.440	0.215	0.644	0.669	0.429	0.003^∗^
D5% (Gy)	0.805	< 0.001^∗^	0.016^∗^	0.081	0.011	0.114	0.022^∗^	0.042^∗^	0.015^∗^	0.475

*B-P*										
V_5Gy_	0.006	0.006^∗^	0.432	0.059	0.027	0.084	0.294	0.026^∗^	0.041^∗^	0.109^∗^
V_10Gy_	0.045	0.045^∗^	0.437	0.169	0.080	0.745	0.553	0.328	0.780	0.300
V_15Gy_	0.749	0.133	0.439	0.202	0.084	0.481	0.203	0.989	0.498	0.354
V_20Gy_	0.175	0.915	0.446	0.209	0.001	0.281	0.144	0.488	0.231	0.367
V_25Gy_	0.536	0.064	0.463	0.215	0.544	0.128	0.108	0.244	0.122	0.365
V_30Gy_	0.016^∗^	0.010	0.508	0.233	0.134	0.133	0.077	0.126	0.068	0.358
V_35Gy_	0.011^∗^	0.011	0.627	0.287	0.044	0.090	0.061	0.066	0.038^∗^	0.351
V_40Gy_	0.010^∗^	0.010	0.929	0.517	0.058	0.057	0.053	0.028^∗^	0.023^∗^	0.431
V_45Gy_	0.005^∗^	0.001^∗^	0.370	0.621	0.056	0.044^∗^	0.085	0.110^∗^	0.029^∗^	0.842
V_50Gy_	0.004^∗^	0.005^∗^	0.002^∗^	0.242	0.179	0.126	0.246	0.029^∗^	0.074	0.458

*Delivery efficiency*										
Mu	< 0.001^∗^	0.001^∗^	< 0.001^∗^	0.002	< 0.001^∗^	< 0.001^∗^	< 0.001^∗^	0.007	0.003	< 0.001^∗^
Time (s)	< 0.001^∗^	0.001^∗^	< 0.001^∗^	0.001	< 0.001^∗^	< 0.001^∗^	< 0.001^∗^	0.007	0.003	< 0.001^∗^

^∗^Indicates statistical significance.

**TABLE 6:** *p* values for multiple comparisons using paired‐samples *t* test (large‐volume subgroups).

Parameter	CRT vs. IMRT	CRT vs. VMAT	CRT vs. CRT + IMRT	CRT vs. CRT + VMAT	IMRT vs. VMAT	IMRT vs. CRT + IMRT	IMRT vs. CRT + VMAT	VMAT vs. CRT + IMRT	VMAT vs. CRT + VMAT	CRT + IMRT vs. CRT + VMAT
*PTV*										
D_min_	0.927	0.892	0.806	0.408	0.873	0.774	0.494	0.935	0.447	0.227
D_max_	0.300	0.508	0.316	0.006^∗^	0.021^∗^	0.102	0.010^∗^	0.531	0.030^∗^	0.619
D_median_	0.743	0.275	0.000^∗^	0.162	0.566	0.339	0.427	0.015^∗^	0.423	0.277
CI	0.001^∗^	0.002^∗^	0.002^∗^	0.005^∗^	0.391	0.022^∗^	0.954	0.014^∗^	0.186	0.106
HI	0.816	0.896	0.254	0.078	0.979	0.098	0.098	0.641	0.045	0.133
V_105%_	0.281	0.060	0.093	0.029^∗^	0.238	0.572	0.183	0.463	0.174	0.238

*CL lung*										
D_max_	0.440	0.565	0.419	0.728	0.678	0.228	0.395	0.253	0.528	0.412
D5% (Gy)	0.488	0.645	0.913	0.604	0.514	0.094	0.386	0.139	0.559	0.199

*Lungs*										
V_30Gy_	0.115	0.278	0.004^∗^	0.084	0.832	0.215	0.142	0.418	0.327	0.145
V_20Gy_	0.501	0.256	0.006^∗^	0.385	0.429	0.354	0.433	0.182	0.209	0.038^∗^
V_10Gy_	0.017^∗^	0.002^∗^	0.010^∗^	0.117	0.154	0.011^∗^	0.017^∗^	0.002^∗^	0.002^∗^	0.016^∗^
V_5Gy_	0.005^∗^	0.001^∗^	0.008^∗^	0.031^∗^	0.076	0.004^∗^	0.008^∗^	0.001^∗^	0.004^∗^	0.014^∗^

IL lung										
V_30Gy_	0.114	0.275	0.003^∗^	0.077	0.833	0.218	0.144	0.418	0.326	0.139
V_20Gy_	0.488	0.252	0.005^∗^	0.372	0.433	0.346	0.419	0.179	0.204	0.041^∗^
V_10Gy_	0.018^∗^	0.016^∗^	0.010^∗^	0.107	0.319	0.012^∗^	0.018^∗^	0.012^∗^	0.014^∗^	0.016^∗^
V_5Gy_	0.001^∗^	0.011^∗^	0.008^∗^	0.035^∗^	0.140	0.001^∗^	0.001^∗^	0.009^∗^	0.010^∗^	0.015^∗^

*Heart*										
V_30Gy_	0.001^∗^	0.048^∗^	0.001^∗^	0.015^∗^	0.642	0.002^∗^	0.004^∗^	0.093	0.061	0.417
V_20Gy_	0.204	0.599	0.001^∗^	0.105	0.378	0.333	0.314	0.471	0.485	0.427
V_10Gy_	0.061	0.071	0.029^∗^	0.915	0.285	0.047^∗^	0.080	0.065	0.062	0.392

*Esophagus*										
D_mean_	0.111	0.006^∗^	0.008^∗^	0.068	0.022^∗^	0.096	0.800	0.005^∗^	0.069	0.058

*Spinal cord*										
D_max_	0.039^∗^	0.009^∗^	0.003^∗^	0.110	0.012^∗^	0.025^∗^	0.151	0.008^∗^	0.305	0.106

*CL breast*										
D_mean_	0.046	0.214	0.286	0.056	0.565	0.032^∗^	0.391	0.186	0.330	0.028^∗^
D5% (Gy)	0.005	0.002	0.394	0.178	0.362	0.343	0.635	0.276	0.395	0.073

*Skin*										
D_mean_	0.159	0.928	0.006^∗^	0.085	0.443	0.392	0.918	0.184	0.253	0.287
D5% (Gy)	0.219	0.615	0.424	0.485	0.574	0.388	0.447	0.036^∗^	0.033^∗^	0.185

*B-P*										
V_5Gy_	0.003	0.000^∗^	0.081	0.008^∗^	0.075	0.002^∗^	0.003^∗^	0.000^∗^	0.000^∗^	0.001^∗^
V_10Gy_	0.012	0.000^∗^	0.048^∗^	0.273	0.005^∗^	0.003^∗^	0.003^∗^	0.000^∗^	0.000^∗^	0.172
V_15Gy_	0.757	0.009^∗^	0.056	0.062	0.001^∗^	0.209	0.089	0.004^∗^	0.002^∗^	0.566
V_20Gy_	0.046^∗^	0.227	0.054	0.055	0.004^∗^	0.204	0.259	0.055	0.037^∗^	0.444
V_25Gy_	0.016^∗^	0.181	0.046^∗^	0.043^∗^	0.046^∗^	0.044^∗^	0.059	0.523	0.610	0.541
V_30Gy_	0.002^∗^	0.013	0.025^∗^	0.027^∗^	0.419	0.004^∗^	0.006^∗^	0.019^∗^	0.021^∗^	0.768
V_35Gy_	0.002^∗^	0.005^∗^	0.007^∗^	0.012^∗^	0.750	0.003^∗^	0.005^∗^	0.007^∗^	0.007^∗^	0.833
V_40Gy_	0.003^∗^	0.471	0.004^∗^	0.016^∗^	0.471	0.004^∗^	0.016^∗^	0.007^∗^	0.002^∗^	0.402
V_45Gy_	0.005^∗^	0.003^∗^	0.003^∗^	0.012^∗^	0.360	0.010^∗^	0.336	0.011^∗^	0.027^∗^	0.234
V_50Gy_	0.148	0.057	0.066	0.057	0.013	0.275	0.058	0.040^∗^	0.240	0.049^∗^

*Delivery efficiency*										
Mu	0.001^∗^	0.013^∗^	< 0.001^∗^	0.209	0.002^∗^	0.002^∗^	< 0.001^∗^	0.114	0.398	0.120
Time (s)	0.001^∗^	0.014^∗^	< 0.001^∗^	0.205	0.002^∗^	0.002^∗^	< 0.001^∗^	0.111	0.403	0.133

^∗^Indicates statistical significance.

### 3.7. Sensitivity Analysis

After excluding the outlier patient (Case No. 02), the overall ranking and relative advantages of the five techniques remained largely unchanged. The CRT + VMAT and VMAT techniques continued to show superior target coverage and homogeneity, while the differences in low‐dose spread (e.g., V_5_ of the ipsilateral lung) became slightly less pronounced but still followed the same trend. Importantly, no reversal of any major finding was observed. Full data from the sensitivity analysis are provided in Table 7.

**TABLE 7:** *p* values for multiple comparisons using paired‐samples *t* test (sensitivity analysis).

Parameter	CRT vs. IMRT	CRT vs. VMAT	CRT vs. CRT + IMRT	CRT vs. CRT + VMAT	IMRT vs. VMAT	IMRT vs. CRT + IMRT	IMRT vs. CRT + VMAT	VMAT vs. CRT + IMRT	VMAT vs. CRT + VMAT	CRT + IMRT vs. CRT + VMAT
*PTV*										
D_min_	0.166	0.873	0.756	0.127	0.397	0.049^∗^	0.913	0.748	0.218	0.015^∗^
D_max_	0.319	0.921	0.093	0.009^∗^	0.345	0.021^∗^	0.018^∗^	0.114	0.006^∗^	0.998
D_median_	0.287	0.180	0.001^∗^	0.091	0.094	0.092	0.049^∗^	0.772	0.070	0.383
CI	< 0.001^∗^	< 0.001^∗^	< 0.001^∗^	< 0.001^∗^	0.134	0.002^∗^	0.248	< 0.001^∗^	0.509	0.002^∗^
HI	0.259	0.610	0.115	0.017^∗^	0.611	0.943	0.169	0.623	0.027^∗^	0.048^∗^
V_105%_	0.111	0.184	0.020^∗^	0.178	0.867	0.441	0.900	0.486	0.910	0.520

*CL lung*										
D_max_	0.203	0.195	0.953	0.195	0.686	0.462	0.860	0.216	0.856	0.219
D5% (Gy)	0.217	0.189	0.731	0.627	0.548	0.262	0.675	0.089	0.308	0.203

*Lungs*										
V_30Gy_	0.012^∗^	0.035^∗^	0.001^∗^	0.012^∗^	0.360	0.029^∗^	0.015^∗^	0.077	0.043^∗^	0.070
V_20Gy_	0.521	0.427	0.004^∗^	0.262	0.752	0.291	0.410	0.271	0.338	0.019^∗^
V_10Gy_	0.001^∗^	0.001^∗^	0.011^∗^	0.049^∗^	0.363	< 0.001^∗^	0.001^∗^	< 0.001^∗^	< 0.001^∗^	0.009^∗^
V_5Gy_	< 0.001^∗^	< 0.001^∗^	0.003^∗^	0.004^∗^	0.006^∗^	< 0.001^∗^	< 0.001^∗^	< 0.001^∗^	< 0.001^∗^	0.002^∗^

*IL lung*										
V_30Gy_	0.011^∗^	0.034^∗^	0.001^∗^	0.010^∗^	0.359	0.028^∗^	0.015^∗^	0.076	0.043^∗^	0.048^∗^
V_20Gy_	0.503	0.420	0.003^∗^	0.246	0.762	0.282	0.392	0.268	0.331	0.020^∗^
V_10Gy_	0.001^∗^	0.002^∗^	0.009^∗^	0.045^∗^	0.579	0.001^∗^	0.001^∗^	0.001^∗^	0.001^∗^	0.008^∗^
V_5Gy_	< 0.001^∗^	< 0.001^∗^	0.003^∗^	0.004^∗^	0.013^∗^	< 0.001^∗^	< 0.001^∗^	< 0.001^∗^	< 0.001^∗^	0.002^∗^

*Heart*										
V_30Gy_	0.000^∗^	0.001^∗^	0.018^∗^	0.014^∗^	0.598	0.000^∗^	0.000^∗^	0.002^∗^	0.001^∗^	0.180
V_20Gy_	0.042^∗^	0.698	0.037^∗^	0.040^∗^	0.454	0.110	0.111	0.936	0.976	0.304
V_10Gy_	0.016^∗^	0.018^∗^	0.088	0.886	0.076	0.011^∗^	0.026^∗^	0.013^∗^	0.016^∗^	0.049^∗^

*Esophagus*										
D_mean_	0.048^∗^	0.000^∗^	0.004^∗^	0.004^∗^	0.000^∗^	0.038^∗^	0.298	0.000^∗^	0.001^∗^	0.003^∗^

*Spinal cord*										
D_max_	0.006^∗^	0.001^∗^	0.068	0.015^∗^	0.001^∗^	0.001^∗^	0.027^∗^	0.001^∗^	0.042^∗^	0.012^∗^

*CL breast*										
D_mean_	0.003^∗^	0.021^∗^	0.125	0.005^∗^	0.248	0.003^∗^	0.784	0.016^∗^	0.210	0.002^∗^
D5% (Gy)	0.001^∗^	0.000^∗^	0.426	0.108	0.204	0.055	0.221	0.035^∗^	0.058	0.004^∗^

*Skin*										
D_mean_	0.468	0.413	0.577	0.459	0.594	0.157	0.723	0.988	0.595	0.033^∗^
D5% (Gy)	0.342	0.835	0.440	0.527	0.780	0.397	0.479	0.003^∗^	0.001^∗^	0.118

*B-P*										
V_5Gy_	< 0.001^∗^	< 0.001^∗^	0.556	0.032^∗^	0.004^∗^	0.002^∗^	0.003^∗^	< 0.001^∗^	< 0.001^∗^	< 0.001^∗^
V_10Gy_	0.002^∗^	< 0.001^∗^	0.554	0.402	0.001^∗^	0.199	0.264	0.024^∗^	0.059	0.057
V_15Gy_	0.933	0.007^∗^	0.551	0.556	0.001^∗^	0.522	0.527	0.390	0.396	0.962
V_20Gy_	0.051	0.492	0.551	0.570	0.003^∗^	0.197	0.209	0.780	0.796	0.607
V_25Gy_	0.098	0.026^∗^	0.580	0.590	0.940	0.036^∗^	0.040^∗^	0.212	0.219	0.771
V_30Gy_	< 0.001^∗^	< 0.001^∗^	0.690	0.684	0.951	0.027^∗^	0.028^∗^	0.038^∗^	0.038^∗^	0.882
V_35Gy_	< 0.001^∗^	< 0.001^∗^	0.987	0.973	0.083	0.009^∗^	0.010^∗^	0.007^∗^	0.007^∗^	0.866
V_40Gy_	< 0.001^∗^	< 0.001^∗^	0.393	0.258	0.052	0.004^∗^	0.008^∗^	0.002^∗^	0.002^∗^	0.260
V_45Gy_	< 0.001^∗^	< 0.001^∗^	0.019^∗^	0.009^∗^	0.049^∗^	0.005^∗^	0.049^∗^	0.001^∗^	0.010^∗^	0.061
V_50Gy_	0.011^∗^	0.002^∗^	0.004^∗^	0.013^∗^	0.013^∗^	0.816	0.855	0.047^∗^	0.073	0.949

*Delivery efficiency*										
Mu	< 0.001^∗^	< 0.001^∗^	< 0.001^∗^	0.082	< 0.001^∗^	< 0.001^∗^	< 0.001^∗^	0.007^∗^	0.034^∗^	0.003^∗^
Time (s)	< 0.001^∗^	< 0.001^∗^	< 0.001^∗^	0.086	< 0.001^∗^	< 0.001^∗^	< 0.001^∗^	0.007^∗^	0.033^∗^	0.003^∗^

^∗^Indicates statistical significance.

## 4. Discussion

This study presents a comprehensive dosimetric comparison of five distinct radiotherapy techniques for left‐sided breast cancer following breast‐conserving surgery. The principal finding is that no single technique is superior across all dosimetric parameters; instead, each modality presents a unique profile of advantages and trade‐offs, particularly between target quality, OAR sparing, and delivery efficiency. This nuanced understanding is crucial for individualized treatment planning.

### 4.1. Synthesis of Key Dosimetric Trade‐Offs

Our results highlight a consistent and critical trade‐off between high‐ and low‐dose exposure, a finding with direct clinical implications. For the heart and lungs, conventional CRT delivered the lowest low‐dose volumes (e.g., heart V_10Gy_, lung V_5Gy_) but resulted in significantly higher high‐dose volumes (e.g., heart V_30Gy_, lung V_30Gy_). In contrast, the modulated techniques (IMRT, VMAT) and hybrid plans excelled at reducing these high‐dose volumes, which are strongly linked to specific clinical toxicities—cardiac perfusion defects and radiation pneumonitis, respectively [16, 17]. However, this benefit came at the cost of a more extensive low‐dose bath, the long‐term clinical impact of which is an area of ongoing investigation, particularly concerning the risk of secondary malignancies [18]. This fundamental trade‐off necessitates a patient‐specific approach, where a patient’s individual anatomy and risk profile (e.g., pre‐existing cardiac or pulmonary conditions) should guide the choice of technique.

Regarding target coverage, all advanced techniques provided superior conformity (CI) compared to CRT. Notably, the hybrid technique CRT + VMAT consistently achieved the most homogeneous dose distributions (HI), suggesting its potential value for complex target volumes where dose uniformity is paramount.

### 4.2. Clinical Interpretation and Technique Selection

The clinical applicability of our findings can be summarized as follows:

• CRT may be considered in select cases where minimizing low‐dose exposure is the highest priority, despite its poorer target conformity and higher intermediate‐/high‐dose to proximal OARs. Its efficiency in delivery remains an advantage.
• IMRT offers excellent target conformity and is highly effective at sparing the heart and lungs from intermediate/high doses. However, its clinical utility is tempered by its high MU requirements and the creation of a significant low‐dose bath.
• VMAT provides a balanced profile, matching IMRT in high‐dose OAR sparing and target quality while offering superior delivery efficiency (fewer MUs, shorter treatment time). Its main drawback is the most extensive low‐dose distribution.
• Hybrid techniques (CRT + IMRT and CRT + VMAT) represent a compelling middle ground. They mitigate the extreme low‐dose bath of IMRT/VMAT while improving upon the high‐dose spill of CRT. CRT + VMAT, in particular, emerged as a robust option, combining the efficient delivery characteristics of VMAT with superior target homogeneity.
• Our findings indicate that PTV volume is a significant modifier of technique performance for certain OARs. This suggests that treatment planning should consider not only the technique but also the patient’s individual anatomy.

### 4.3. Comparison With Existing Literature

Our findings both corroborate and refine the existing body of evidence. The conclusion by Sas‐Korczyńska et al. [11] and Mo et al. [19] that IMRT and VMAT improve target homogeneity and reduce high‐dose OAR exposure compared to CRT is strongly supported by our data. Furthermore, our observation that VMAT enhances delivery efficiency aligns with the findings of Yu et al. [20]. However, our more granular analysis of hybrid techniques provides a critical extension to this work. While Bi et al. [10] favored hybrid IMRT for right‐sided breast cancer with a simultaneous integrated boost, our results for left‐sided disease demonstrate that hybrid VMAT (CRT + VMAT) is highly competitive, offering an optimal balance of dosimetric quality and efficiency. This discrepancy underscores that the optimal technique may be influenced by laterality, the use of a boost, and specific planning objectives.

### 4.4. Limitations

This study has several limitations. First, the sample size is small (only 10 patients), which limits statistical power and generalizability; therefore, the findings should be interpreted as preliminary and validated in larger cohorts. Furthermore, one patient had an unusually small PTV (250.9 cc) due to Stage 0 ductal carcinoma in situ. Although sensitivity analysis excluding this case did not alter the main conclusions, the small sample size (*n* = 10) combined with this outlier warrants caution when generalizing the findings. Future studies with larger and more diverse patient populations are needed to confirm the robustness of the observed technique rankings. Second, pairwise *t* tests were performed without adjustment for multiple comparisons (e.g., Bonferroni). Given the exploratory nature of this dosimetric planning study and the small sample size, *p* values between 0.01 and 0.05 should be interpreted as indicating trends rather than definitive differences. Future confirmatory studies with larger samples should apply appropriate corrections. Third, as a dosimetric planning study, it does not report clinical outcomes such as toxicity rates or survival. The potential clinical impact of the observed low‐dose differences remains to be validated in long‐term patient follow‐up studies. Fourth, no low‐dose constraints (e.g., V_5_, V_10_) were applied during optimization. Therefore, the larger low‐dose bath observed for IMRT and VMAT may reflect optimization choices rather than inherent technical limitations. In clinical practice, adding low‐dose penalties could reduce low‐dose spread, but this might compromise target conformity or high‐dose OAR sparing. Our findings represent a comparison under standard, low‐dose‐unconstrained conditions, which are common in real‐world planning. Fifth, jaw tracking was not used in this study. Jaw tracking reduces low‐dose volumes (e.g., V_5_, V_10_) by minimizing interleaf leakage and out‐of‐field scatter. Because it was disabled, the low‐dose bath for IMRT and VMAT may be overestimated compared to clinical practice where jaw tracking is enabled. Thus, our findings reflect a jaw‐tracking‐disabled scenario, and the low‐dose differences may not be directly generalizable to centers that routinely use jaw tracking. Future studies should assess whether jaw tracking affects the relative performance of different techniques for low‐dose endpoints. Furthermore, the planning approach was standardized, and variations in planner skill or specific planning system algorithms could influence the results. Finally, patient‐specific factors such as extremely large breast size or unusual anatomy were not explored in subgroups.

## 5. Conclusion and Clinical Implications

In conclusion, the optimal selection of radiotherapy technique for left‐sided breast cancer should be guided by individualized clinical priorities, as no single approach demonstrates absolute dosimetric superiority across all parameters. For cases where superior target coverage and homogeneity are paramount, the CRT + VMAT plan represents a balanced and versatile choice. When enhanced sparing of the heart and lungs, with particular emphasis on intermediate‐to high‐dose levels, is critically needed, IMRT or CRT + IMRT may be preferred. Conversely, when delivery efficiency and minimization of low‐dose bath are prioritized, CRT remains a relevant option. The integration of modern auxiliary technologies, such as respiratory gating and real‐time image guidance, can further refine the implementation of these techniques [21–24]. Future studies should explore their application within hypofractionated regimens, which may amplify dosimetric advantages while shortening treatment courses, thereby enriching the evidence base for personalized radiotherapy strategy.

## Funding

This work was funded by the Health Commission of Hebei Province, Grant No. 20181196.

## Ethics Statement

The authors have nothing to report.

## Conflicts of Interest

The authors declare no conflicts of interest.

## Data Availability

The data that support the findings of this study are available on request from the corresponding author. The data are not publicly available due to privacy or ethical restrictions.
